# Nurse-performed screening for postextubation dysphagia: a retrospective cohort study in critically ill medical patients

**DOI:** 10.1186/s13054-016-1507-y

**Published:** 2016-10-12

**Authors:** Kay Choong See, Si Yu Peng, Jason Phua, Chew Lai Sum, Johncy Concepcion

**Affiliations:** 1 Division of Respiratory & Critical Care Medicine, University Medicine Cluster, National University Health System, 1E Kent Ridge Road, NUHS Tower Block Level 10, Singapore, 119228 Singapore; 2 Yong Loo Lin School of Medicine, National University of Singapore, Singapore, Singapore; 3 Department of Nursing, National University Hospital, Singapore, Singapore; 4 Department of Rehabilitation, National University Hospital, Singapore, Singapore

**Keywords:** Airway extubation, Deglutition disorders, Intensive care unit, Intubation, Pneumonia

## Abstract

**Background:**

Swallowing difficulties are common, and dysphagia occurs frequently in intensive care unit (ICU) patients after extubation. Yet, no guidelines on postextubation swallowing assessment exist. We aimed to investigate the safety and effectiveness of nurse-performed screening (NPS) for postextubation dysphagia in the medical ICU.

**Methods:**

We conducted a retrospective cohort study of mechanically ventilated patients who were extubated in a 20-bed medical ICU. Phase I (no NPS, October 2012 to January 2014) and phase II (NPS, February 2014 to July 2015) were compared. In phase II, extubated patients received NPS up to three times on consecutive days; patients who failed were referred to speech-language pathologists. Outcomes analyzed included oral feeding at ICU discharge, reintubation, ICU readmission, postextubation pneumonia, ICU and/or hospital mortality, and ICU and/or hospital length of stay (LOS). Subgroup analysis was done for patients extubated after >72 h of mechanical ventilation, as the latter may predispose patients to postextubation dysphagia. Multivariable adjustments for Acute Physiology and Chronic Health Evaluation (APACHE) II score and comorbidities were done because of baseline differences between the phases.

**Results:**

A total of 468 patients were studied (281 in phase I, 187 in phase II). Patients in phase II had higher APACHE II scores than those in phase I (27.2 ± 8.2 vs. 25.4 ± 8.2; *P* = 0.018). Despite this, patients in phase II showed a 111 % increase in (the odds of) oral feeding at ICU discharge and a 59 % decrease in postextubation pneumonia (multivariate *P* values 0.001 and 0.006, respectively). In the subgroup analysis, NPS was associated with a 127 % increase in oral feeding at ICU discharge, an 80 % decrease in postextubation pneumonia, and a 25 % decrease in hospital LOS (multivariate *P* values 0.021, 0.004, and 0.009, respectively). No other outcome differences were found.

**Conclusions:**

NPS for dysphagia is safe and may be superior to no screening with respect to several patient-centered outcomes.

**Electronic supplementary material:**

The online version of this article (doi:10.1186/s13054-016-1507-y) contains supplementary material, which is available to authorized users.

## Background

Swallowing difficulties are common, and dysphagia occurs in up to 62 % of intensive care unit (ICU) patients after extubation [1]. Postextubation dysphagia may be related to several mechanisms: (1) impaired strength and sensation of the tongue [2]; (2) laryngeal damage [3, 4]; (3) neuromuscular impairment [5]; and (4) cognitive complications of critical illness, such as somnolence and sedation, contributing to discoordination of the swallowing reflex [6]. In turn, postextubation dysphagia is associated with delayed oral food intake (potentially increasing malnutrition and dehydration) and increased hospital-acquired pneumonia, reintubation, hospitalization duration, and mortality [5, 7–9]. This is particularly important in elderly ICU patients because of delayed swallowing, defective larynx elevation, and loss of cough efficacy [10, 11]. Accurate assessment of swallowing is therefore important and often formally undertaken by speech-language pathologists (SLPs).

Yet, no guidelines on postextubation swallowing assessment exist [12]. Also, postextubation swallowing assessment is not done regularly, as only 41 % of hospitals were found to routinely screen extubated patients for dysphagia in one study, and only 44 % of patients completed a swallowing assessment in another study [7, 12]. Similarly, in our institution, prior to this study, patients were not receiving accurate screening postextubation, and screening for postextubation dysphagia could be improved.

One possible method of making postextubation dysphagia assessment more streamlined and consistent would be to invite nurses to perform bedside swallow screening for postextubation patients. Patients who pass the screening would then be quickly put on an oral diet, which is an important psychological boost [13]. Conversely, patients who failed the screening would then be referred to SLPs for early management, with potentially favorable outcomes [6].

However, few data exist on the safety (i.e., pneumonia rates, reintubation rates, mortality, hospitalization duration) and effectiveness (i.e., oral feeding rates) of dysphagia screening performed by professionals other than SLPs, especially in nonneurological patient cohorts [7, 12]. One protocol (described by Massey) involved a 60-ml water swallow test and was implemented by SLPs, but only 25 poststroke patients were studied, and safety information was not available [14]. Another protocol (described by Leder) involved a 90-ml water swallow test and could be implemented by either SLPs or nurses, but the results showed poor specificity (about half the patients who failed the test were actually able to safely feed orally) [15, 16]. A third protocol, the Toronto Bedside Swallowing Screening Test, has been developed for stroke inpatients [17], but it has not been validated for postextubation patients. We believe that accurate dysphagia screening for postextubation patients with mixed etiologies could be done by health professionals other than SLPs. Therefore, we sought to audit the practice of nurse-performed screening (NPS) for postextubation dysphagia in our medical ICU.

## Methods

### Study population and outcomes

We performed a retrospective cohort study of all patients extubated in our 20-bed medical ICU between October 2012 and July 2015. Information was drawn from the electronic medical records, which routinely state the patient’s premorbid condition, including the ability to feed orally. Only the first extubation for each patient during the 3-year study period was analyzed, as one of our outcomes studied was reintubation. Patients did not require any minimal duration of intubation to be included in this study, and there were no age limits. Also, we did not have any patients who were intubated only for procedures. We excluded patients who were unable to feed orally before their critical illness, those who developed a permanent condition that precluded oral feeding as determined by the managing ICU team (e.g., major brainstem stroke), those who required continuous noninvasive ventilation for more than 6 h after extubation, those who were terminally extubated, or those who had undergone tracheostomy [15]. Patients who did not pass the NPS (e.g., those who failed the first screening but were transferred out of the ICU before the second and third screenings) were still included in the analysis.

In phase I (October 2012 to January 2014), patients did not receive NPS for dysphagia; in phase II (February 2014 to July 2015), patients received NPS. The duration of phase II was determined such that we could obtain at least two-thirds as many patients as we did for phase I: 148 patients in phase II would be required to detect an absolute increase in oral feeding at ICU discharge from 61.6 % to 76.6 %, with power set at 80 % and the significance level set at 5 %. This was an important consideration because our inpatient numbers dropped considerably after the opening of a major new hospital only 7.2 km away. Outcomes analyzed included oral feeding at ICU discharge, reintubation, ICU readmission, postextubation pneumonia, ICU and/or hospital mortality, and ICU and/or hospital length of stay (LOS). Patients had to be completely weaned off tube feeding to be counted as being on oral feeding. The presence of postextubation pneumonia was established by the managing physician using clinical and radiological criteria (new onset or, in the case of preexisting pneumonia, worsening of symptoms, oxygenation, and radiology), which are consistent with the hospital-acquired and ventilator-associated pneumonia clinical practice guidelines issued by the Infectious Diseases Society of America and the American Thoracic Society [18]. One of the authors (SYP) screened all the case records for postextubation pneumonia; two of the authors (KCS, JP) who are also pulmonology specialists verified all diagnoses of postextubation pneumonia. We performed a secondary analysis of quality improvement data, the latter being collected to support the effort of empowering nurses to perform dysphagia screening.

### Interventions

Patients were mechanically ventilated using low tidal volume ventilation with minimal analgesia and sedation. No patient was placed on high-frequency oscillatory ventilation [19]. Sepsis was treated with early, broad-spectrum antibiotics and source control. Nasogastric tubes were used for enteral nutrition for intubated patients. Patients who could not be orally fed continued to receive nutrition via nasogastric tubes. No patient received surgically placed feeding tubes or parenteral nutrition while in the ICU.

Postextubation, in phase I, any 1 of 25 pulmonary/critical care physicians, using variable informal methods of assessment, determined if patients could be fed orally. The most popular method was asking patients to take a few sips of water from a straw 1–2 h postextubation, and clinicians assumed that patients could safely feed orally if the patients did not choke. Another method was to ask patients to drink some water directly from a cup. Choking was taken as any visible choking and drooling of water. No other criteria were used to determine swallowing function, and physicians did not retest patients who failed their swallowing screens. SLPs were not consulted by physicians in phase I while the patients were in the ICU. Patients who were not able to feed orally were routinely seen by SLPs after ICU discharge.

In phase II, nurses with at least 3 years of intensive care nursing experience screened patients for dysphagia using the NPS protocol before oral feeding was instituted, with existing nasogastric tubes in situ. (These did not seem to pose significant problems with swallowing assessment.) We used the term *screening* in NPS according to the definition developed by the American Speech-Language-Hearing Association, which is a “pass/fail procedure to identify an individual who may need a complete dysphagia assessment” [20]. However, we did not specifically include methods to assess reliability or sensitivity, because we had adapted a preexisting dysphagia screening test (the Massey Bedside Swallowing Screen [14]), shown in Additional file 1. Each dysphagia screen involved first feeding the patient 5 ml of water by mouth, followed by 60 ml of water. We believed that 60 ml of water would provide an optimal balance of sensitivity and specificity as compared with Leder’s protocol [15, 16]. Nurses would look for signs of aspiration, such as choking and gurgling. Patients who swallowed water without signs of aspiration would then be started on oral feeding according to their dentition status (finely minced diet for edentulous patients and chopped diet for dentate patients), without further SLP evaluation. Documentation would be done in the Dysphagia Screening Form (see Additional file 2).

The first NPS screening would occur on the day of extubation at least 1 h postextubation. Patients who failed the first screen would have a repeat screen the next day between 0600 h and 0900 h. This was done because patients’ swallowing function may recover rapidly [15], though admittedly the recovery may be delayed in elderly ICU patients [11]. Patients who failed the second screen would have a final screen the next day between 0600 h and 0900 h. Patients who failed all three screens or who were transferred to the general floor before passing any screen, would be referred to SLPs for formal swallowing assessment and management. In practice, this meant that SLP referrals were usually not done while patients remained in the ICU after extubation. We allowed attending physicians to override the NPS protocol at their discretion at any time. NPS screens were also not done after patients left the ICU, which meant that not all patients had the opportunity to be screened thrice.

### Training process for nurses

Forty nurses in the ICU were trained to perform dysphagia screening by an SLP with 15 years of experience (JC). For each ICU shift, at least one trained nurse would be available. The nurses each underwent a 3-h classroom-based theory and practice session, which started with a pretest (see Additional file 3), followed by didactic lectures on normal and abnormal swallowing. Nurses then learned and practiced the NPS method on each other. At the end of the classroom session, nurses answered the same pretest items and had to score at least 80 % before proceeding to practical competency testing. At a separate practical test session, using the competency assessment form (see Additional file 4), participants paired up (one acted as a patient, another acted as the screener), and each screener had to successfully perform three screens using the NPS method. All nurses needed to fulfill the same training standard, and we did not measure the interrater reliability of the nurses’ actual assessment of swallowing. While we did not formally test the nurses, we believe that the nurses were able to maintain the fidelity of NPS over time due to constant practice.

### Statistical analysis

Univariate comparisons of proportions, means, and medians were done using Fisher’s exact test, Student’s *t* test, and the Wilcoxon rank-sum test, respectively. Medians were used instead of means for nonnormally distributed variables such as ventilator days and LOS. Multivariable adjustments were done for baseline patient characteristics that significantly differed between phases I and II (i.e. Acute Physiology and Chronic Health Evaluation [APACHE] II score measured within 24 h of ICU admission) and comorbidities. Logistic regression was done for dichotomous outcomes (oral feeding at ICU discharge, reintubation, ICU readmission, postextubation pneumonia, ICU and/or hospital mortality), while linear regression was done for log-transformed ICU and/or hospital LOS (log transformation was done for normality).

Subgroup analysis was done for patients extubated after >72 h of mechanical ventilation because a longer duration of endotracheal intubation may predispose patients to postextubation dysphagia [4, 21–23]. To further evaluate the safety of NPS, we restricted the analysis of postextubation pneumonia to only patients who were allowed oral feeding at the point of transfer from the ICU to the general floor, because this would allow elucidation of any association between oral feeding and postextubation pneumonia. To verify that the NPS was used according to protocol, we studied the results of NPS and the correlation between the NPS and oral feeding at ICU discharge. Statistical significance was taken as *P* < 0.05.

## Results

We studied 468 patients (281 in phase I and 187 in phase II) who were ventilated for a median of 2 days prior to extubation. We excluded 104 patients in phase I: 6 who were unable to feed orally before their critical illness, 6 who developed a permanent condition that precluded oral feeding, 69 who were terminally extubated, and 23 who had undergone tracheostomy. In comparison, we excluded 67 patients in phase II: 1 who was unable to feed orally before the critical illness, 2 who developed a permanent condition that precluded oral feeding, 52 who were terminally extubated, and 12 who had undergone tracheostomy. Patients in phase II, compared with those in phase I, were more ill with higher APACHE II scores, more had congestive heart failure as a comorbidity, and more had bronchiectasis as a comorbidity (Table 1). Despite this, proportionally more patients in phase II were allowed oral feeding at ICU discharge, with fewer patients developing pneumonia postextubation (see Additional file 5). In phase I, 11 patients (3.9 %) were reintubated for the following reasons: 6 for hospital-acquired pneumonia, 3 for acute heart failure, 1 for massive gastrointestinal bleeding, and 1 for asystolic collapse of unclear cause. In phase II, 13 patients (7.0 %) were reintubated for the following reasons: 2 for hospital-acquired pneumonia, 2 for extrapulmonary sepsis, 1 for massive malignant pleural effusion, 1 for severe asthma, 5 for acute heart failure, 1 for massive gastrointestinal bleeding, and 1 for status epilepticus. No significant differences in reintubation, ICU readmission, ICU and/or hospital mortality, or ICU and/or hospital LOS were found (Table 2).

**Table 1:** Patient characteristics

Characteristics	All patients (*n* = 468)	Phase I (*n* = 281)	Phase II (*n* = 187)	*P* value
Age, years	60.4 ± 16.2	59.7 ± 17.7	61.4 ± 13.5	0.255
Female sex, *n* (%)	172 (36.8)	103 (36.7)	69 (36.9)	1.000
APACHE II score	26.1 ± 8.3	25.4 ± 8.2	27.2 ± 8.2	0.018*
Weight, kg	63.7 ± 17.8	63.7 ± 17.8	63.6 ± 18.0	0.946
Ventilated days preextubation, median (IQR)	2 (1–4)	2 (1–4)	3 (1–5)	0.509
Main diagnosis, *n* (%)				0.085
Pneumonia	130 (27.8)	67 (23.8)	63 (33.7)	
Nonpneumonia sepsis	72 (15.4)	45 (16.0)	27 (14.4)	
COPD	16 (3.4)	7 (2.5)	9 (4.8)	
Asthma	29 (6.2)	16 (5.7)	13 (7.0)	
Fluid overload	32 (6.8)	21 (7.5)	11 (5.8)	
Stroke	11 (2.4)	10 (3.6)	1 (0.5)	
Seizure	30 (6.4)	19 (6.8)	11 (5.9)	
Other^a^	148 (31.6)	96 (34.2)	52 (27.8)	
Comorbidity, *n* (%)				
Diabetes mellitus	184 (39.4)	100 (35.7)	84 (44.9)	0.053
Hypertension	255 (54.6)	151 (53.9)	104 (55.6)	0.776
IHD	111 (23.8)	60 (21.4)	51 (27.3)	0.151
CHF	38 (8.1)	14 (5.0)	24 (12.8)	0.003*
Asthma	66 (14.1)	37 (13.2)	29 (15.5)	0.500
COPD	30 (6.4)	14 (5.0)	16 (8.6)	0.129
Bronchiectasis	8 (1.7)	1 (0.4)	7 (3.7)	0.008*
OSA	23 (4.9)	12 (4.3)	11 (5.9)	0.514
CKD	107 (23.0)	59 (21.2)	48 (25.7)	0.263
CLD	26 (5.6)	18 (6.4)	8 (4.3)	0.411
Stroke	59 (12.7)	40 (14.3)	19 (10.2)	0.204
Cancer	34 (12.1)	24 (12.8)	24 (12.8)	0.886

*Abbreviations: APACHE* Acute Physiology and Chronic Health Evaluation, *CHF* Chronic heart failure, *CKD* Chronic kidney disease, *COPD* Chronic obstructive pulmonary disease, *CLD* Chronic liver disease, *IHD* Ischemic heart disease, *IQR* Interquartile range, *OSA* Obstructive sleep apnea

* *P* < 0.05

^a^ Including hyperglycemic crises, drug overdose, fulminant hepatic failure, major trauma, neuromuscular dysfunction

**Table 2:** Patient outcomes

Outcomes	Phase I (*n* = 281)	Phase II (*n* = 187)	Univariate *P* value	Multivariate ratio^a,b^ (95 % CI)	Multivariate *P* value^a^
Oral feeding on ICU discharge, *n* (%)	173 (61.6)	144 (77.0)	0.001*	2.11 (1.37–3.25)	0.001*
Pneumonia postextubation, *n* (%)	45 (16.1)	15 (8.0)	0.011*	0.41 (0.22–0.77)	0.006*
Reintubation, *n* (%)	11 (3.9)	13 (7.0)	0.198	1.65 (0.71–3.87)	0.246
Readmission to ICU, *n* (%)	15 (5.3)	19 (10.2)	0.068	1.72 (0.83–3.55)	0.145
ICU mortality, *n* (%)	3 (1.1)	4 (2.1)	0.445	2.16 (0.47–9.91)	0.320
Hospital mortality, *n* (%)	19 (6.8)	13 (7.0)	1.000	0.87 (0.41–1.86)	0.726
ICU LOS, days, median (IQR)	6 (4–8)	6 (4–9)	0.170	1.07 (0.95–1.21)	0.247
Hospital LOS, days, median (IQR)	16 (9–27)	14 (9–22)	0.170	0.87 (0.75–1.00)	0.058

*Abbreviations: ICU* Intensive care unit, *IQR* Interquartile range, *LOS* Length of stay

* *P* < 0.05

^a^ Adjusted for Acute Physiology and Chronic Health Evaluation II score, congestive heart failure (as a comorbidity), and bronchiectasis (as a comorbidity) using logistic regression (for dichotomous outcomes) and using multiple linear regression (for log-transformed LOS)

^b^ Odds ratio for logistic regression (for dichotomous outcomes) and exponentiated ratio for multiple linear regression (for log-transformed LOS)

When we restricted the analysis to patients extubated after >72 h of mechanical ventilation, we found that phase I and phase II patients were not significantly different (Table 3). Again, we found that proportionally more patients in phase II were allowed oral feeding at ICU discharge, with fewer patients developing pneumonia postextubation (see Additional file 5). Furthermore, patients in phase II, compared with those in phase I, had decreased hospital LOS. No other outcome differences were found (Table 4).

**Table 3:** Subgroup analysis of characteristics of patients extubated after >72 h of mechanical ventilation

Characteristics	All patients (*n* = 160)	Phase I (*n* = 94)	Phase II (*n* = 66)	*P* value
Age, years	58.5 ± 15.9	58.4 ± 17.4	58.7 ± 13.7	0.879
Female sex, *n* (%)	59 (36.9)	35 (37.2)	24 (36.4)	1.000
APACHE II score	27.6 ± 8.9	26.7 ± 8.9	29.0 ± 8.7	0.111
Weight, kg	67.0 ± 19.8	68.0 ± 21.4	65.7 ± 17.3	0.472
Ventilator days preextubation	6 (4–8)	6 (4–9)	6 (4–8)	0.807
Main diagnosis, *n* (%)				0.128
Pneumonia	53 (33.1)	27 (28.7)	26 (39.4)	
Nonpneumonia sepsis	21 (13.1)	10 (10.6)	11 (16.7)	
COPD	6 (3.8)	2 (2.1)	4 (6.1)	
Asthma	9 (5.6)	5 (5.3)	4 (6.1)	
Fluid overload	13 (8.1)	8 (8.5)	5 (7.6)	
Stroke	6 (3.8)	6 (6.4)	0 (0.0)	
Seizure	10 (6.3)	8 (8.5)	2 (3.0)	
Other^a^	42 (26.3)	28 (29.8)	14 (21.2)	
Comorbidity, *n* (%)				
Diabetes mellitus	66 (41.5)	34 (36.6)	32 (48.5)	0.145
Hypertension	86 (54.1)	51 (54.8)	35 (53.0)	0.872
IHD	41 (25.8)	25 (26.9)	16 (24.2)	0.854
CHF	8 (5.0)	3 (3.2)	5 (7.6)	0.278
Asthma	23 (14.5)	11 (11.8)	12 (18.2)	0.360
COPD	8 (5.0)	2 (2.2)	6 (9.1)	0.067
Bronchiectasis	3 (1.9)	0 (0.0)	3 (4.6)	0.070
OSA	11 (6.9)	7 (7.5)	4 (6.1)	1.000
CKD	36 (22.6)	18 (19.4)	18 (27.3)	0.254
CLD	6 (3.8)	4 (4.3)	2 (3.0)	1.000
Stroke	19 (12.0)	14 (15.1)	5 (7.6)	0.215
Cancer	17 (10.7)	9 (9.7)	8 (12.1)	0.615

*Abbreviations: APACHE* Acute Physiology and Chronic Health Evaluation, *CHF* Chronic heart failure, *CKD* Chronic kidney disease, *COPD* Chronic obstructive pulmonary disease, *CLD* Chronic liver disease, *IHD* Ischemic heart disease, *IQR* Interquartile range, *OSA* Obstructive sleep apnea

^a^ Including hyperglycemic crises, drug overdose, fulminant hepatic failure, major trauma, neuromuscular dysfunction

**Table 4:** Subgroup analysis of outcomes for patients extubated after >72 h of mechanical ventilation

Outcomes	Phase I (*n* = 94)	Phase II (*n* = 66)	Univariate *P* value	Multivariate ratio^a,b^ (95 % CI)	Multivariate *P* value^a^
Oral feeding on ICU discharge, *n* (%)	46 (48.9)	44 (66.7)	0.035*	2.27 (1.13–4.54)	0.021*
Pneumonia postextubation, *n* (%)	24 (25.5)	5 (7.6)	0.004*	0.20 (0.07–0.60)	0.004*
Reintubation, *n* (%)	6 (6.4)	9 (13.6)	0.168	1.80 (0.58–5.62)	0.306
Readmission to ICU, *n* (%)	7 (7.5)	10 (15.2)	0.128	1.79 (0.62–5.18)	0.285
ICU mortality, *n* (%)	1 (1.1)	3 (4.6)	0.307	4.62 (0.46–46.2)	0.192
Hospital mortality, *n* (%)	7 (7.5)	6 (9.1)	0.773	0.96 (0.29–3.18)	0.944
ICU LOS, days, median (IQR)	9 (7–13)	9 (7–12)	0.704	0.97 (0.83–1.13)	0.672
Hospital LOS, days, median (IQR)	24 (17–39)	18 (12–30.5)	0.010*	0.75 (0.61–0.93)	0.009*

*Abbreviations: CI* Confidence interval, *ICU* Intensive care unit, *IQR* Interquartile range, *LOS* Length of stay

* *P* < 0.05

^a^ Adjusted for Acute Physiology and Chronic Health Evaluation II score, congestive heart failure (as a comorbidity) and bronchiectasis (as a comorbidity), using logistic regression (for dichotomous outcomes) and using multiple linear regression (for log-transformed LOS)

^b^ Odds ratio for logistic regression (for dichotomous outcomes), and exponentiated ratio for multiple linear regression (for log-transformed LOS)

The overall safety signal favored NPS even when we analyzed only patients who were allowed oral feeding at the point of transfer from the ICU to the general floor. Among all patients, 19 of 173 in phase I and 10 of 144 patients in phase II developed pneumonia postextubation (11.0 % vs. 6.9 %, *P* = 0.244). In this analysis, when we considered only patients who were extubated after >72 h of mechanical ventilation, 9 of 46 patients in phase I and 3 of 44 patients in phase II developed pneumonia postextubation (19.6 % vs. 6.8 %, *P* = 0.120).

Regarding NPS use, 98.9 % of patients received at least one NPS screen. Among patients who passed the swallowing screen, 142 (99.3 %) of 143 did so by the second screen (Table 5). Of 44 patients who failed NPS screening, 38 (86.4 %) were discharged from the ICU before three screens could be completed. Overall correlation between the swallowing screen result and oral feeding status on ICU discharge was good at 92.0 % (see Additional file 6): 136 (95.1 %) of 143 patients who passed the screen were allowed oral feeding by their attending physicians, while 36 (81.8 %) of 44 patients who failed the screen were not allowed oral feeding. In other words, 8 % of nursing recommendations were overridden by a physician.

**Table 5:** Results of swallowing screening (*N* = 187 patients)

Swallowing screen	Number of patients	Passed, *n* (%)	Failed, *n* (%)	Missed, *n* (%)
Day 1 result only	187	115 (61.5)	67 (35.8)	5^a^ (2.7)
Day 2 result only	73	28 (38.4)	16 (21.9)	29^b^ (39.7)
Day 3 result only	44	1 (2.3)	6 (13.6)	37^c^ (84.1)
Overall result	187	143^d^ (76.5)	42^e^ (22.5)	2^f^ (1.1)

^a^ Missed the swallowing screen on day 1

^b^ Missed the swallowing screen on day 2

^c^ Missed the swallowing screen on day 3

^d^ One patient who passed on day 1, had a repeat test of day 2 and passed

^e^ Did not pass the swallowing screen and had at least 1 day of screening

^f^ Missed all 3 days of screening

## Discussion

NPS for dysphagia, compared with no NPS, was associated with a 111 % increase in (the odds of) oral feeding at ICU discharge and a 59 % decrease in postextubation pneumonia. Among patients extubated after >72 h of mechanical ventilation, NPS for dysphagia, compared with no NPS, was associated with a 127 % increase in oral feeding at ICU discharge, an 80 % decrease in postextubation pneumonia, and a 25 % decrease in hospital LOS. We also found relatively few instances of attending physicians overriding the NPS protocol. These results suggest that NPS is safe, likely to be superior to usual care without NPS, and acceptable to medical teams.

Our study has validated the safety of adapting the Massey Bedside Swallowing Screen for use by nurses [14]. The results are strengthened by NPS being associated with decreased rates of postextubation pneumonia, even though our nonrandomized study design resulted in NPS patients being more ill overall (as demonstrated by APACHE II score differences). The association of NPS with decreased postextubation pneumonia was also seen in patients who did *not* have pneumonia as their main diagnosis (OR 0.42, 95 % CI 0.19–0.92, *P* = 0.029), after adjustment for APACHE II score, congestive heart failure (as a comorbidity), and bronchiectasis (as a comorbidity). We postulate that NPS, compared with no NPS, could improve oral feeding rates because patients were allowed additional screening after failing the first one. We additionally postulate that NPS, compared with no NPS, could better identify patients at risk of aspiration. The smaller proportion of patients with stroke or seizures in phase II could not explain the association of NPS with decreased postextubation pneumonia. Of the 29 patients with stroke and/or seizure in phase I, 6 (20.7 %) developed postextubation pneumonia. In comparison, of 12 patients with stroke and/or seizure in phase II, 2 (16.7 %) developed postextubation pneumonia, which is a nonsignificant difference (*P* = 1.000 by Fisher’s exact test). The imbalance of patients with stroke also did not significantly influence the incidence of postextubation dysphagia. We did an analysis excluding these patients, and the difference in oral feeding rates remained statistically significant (168 [66.1 %] of 254 in phase I vs. 135 [77.1 %] of 175 in phase II; *P* = 0.017).

Separately, the association of NPS with decreased hospital LOS in patients who were extubated after prolonged mechanical ventilation could be due to fewer patients being fed nonorally at ICU discharge. Presumably, nonoral feeding may delay hospital discharge because more time is required for the transition to oral feeding or for training caregivers to administer nutrition nonorally (usually via nasogastric feeding in our setting).

Although it was not statistically significant, we cannot completely dismiss a possible association between NPS screening and reintubation, the latter accounting for the majority of ICU readmissions. However, reintubation events appear to be due to causes other than pneumonia. Of the 13 patients in phase II who were reintubated, only 2 were reintubated because of postextubation pneumonia, with the rest being reintubated because of new-onset nonpneumonia sepsis, fluid overload, or neurological deficits. Because the reasons for reintubation were varied in phase II, we feel that the slightly higher rate of reintubation was a chance finding.

Some limitations exist in our study. First, we conducted this research with medical ICU patients, and the results may not be generalizable to surgical or cardiothoracic ICU patients. The 36 % prevalence of dysphagia on day 1 postextubation may seem high, and few comparable data are available for short mechanical ventilation duration in medical ICU patients, though there is one paper showing a dysphagia rate of 17 % for intubation durations of 24–48 h among patients following cardiovascular surgery [23]. Second, we did not verify the screening results of NPS using instrumental techniques such as flexible endoscopic evaluation of swallowing and videofluoroscopic swallow studies. In particular, NPS would not be able to detect silent aspiration (i.e., aspirating without a protective cough response), unlike instrumental techniques. However, the validity of NPS is now supported by improved pneumonia rates and hospitalization durations, while instrumental evaluation has not been shown to affect patient outcomes [12, 24, 25]. Third, the timing of the dysphagia screening was set to start within the first day postextubation, though recent data show that the timing does not affect the result of swallowing assessment [26]. Fourth, no patient was kept in the ICU for NPS screening per se, and some patients who failed NPS screening were discharged from the ICU before three screens could be completed. We thus do not know if our results could have been improved if all patients had been able to receive all three NPS screens. Fifth, we acknowledge that physician determination of swallowing function may not be done in some institutions, which limits the generalizability of the results. Sixth, our median ICU LOS was relatively short at 6 days. However, this duration was similar in phases I and II. In order not to miss any postextubation pneumonia, we counted all the cases of postextubation pneumonia that occurred in the ICU or on the general floor subsequently. We also do not think that patients would develop a clinically significant but unidentified postextubation pneumonia after leaving the ICU. Seventh, our study design was not the most scientifically robust; that is, we did a retrospective cohort study rather than a randomized trial. However, it would be difficult to perform blinding and to avoid nurses’ applying dysphagia screening skills within the same ICU, potentially biasing results toward the null.

Current methods of identifying at-risk patients for SLP referral appear to rest on the duration of prior intubation, and a common definition of *prolonged intubation* uses a cutoff of 48 h [4, 6, 27]. However, if we applied this cutoff to our patient population, 50 % of all patients would have needed to be referred, which would incur substantial SLP time and resources. In place of direct SLP referral, our data suggest that screening could be done by nurses first. In our experience, the bedside swallow screen takes only 5–10 minutes and is relatively easy to perform. Nonetheless, further research is needed to check whether NPS, versus no NPS, would result in more expedient and more appropriate referrals to SLPs for swallowing dysfunction (i.e., avoiding both overuse and underuse of SLP resources).

We hope that our study can stimulate further investigation into the development of pragmatic protocols for the assessment of swallowing impairment postextubation. Our protocol appeared to be safe for medical patients and should be validated in other patient cohorts, and using a randomized trial design. Importantly, nurses can be readily trained—as we have described—to implement the NPS protocol. Extension of NPS to the general floor could conceivably be done, either by trained general floor nurses or by more specialized ICU liaison nurses [28]. Finally, cost-effectiveness studies could be done to quantify the resource savings of NPS, which could accrue from lower treatment costs due to less postextubation pneumonia and decreased hospital LOS.

## Conclusions

NPS for postextubation dysphagia is safe and is likely to be superior to no screening with respect to several patient-centered outcomes. Our results should encourage wider adoption and audit of practical protocols that enable bedside nurses to routinely screen for dysphagia in the ICU. We believe that empowering nurses to do so may simultaneously expand their scope of practice and enhance patient care.

## Additional files

Additional file 1: Figure S1. Nurse-performed screening for dysphagia workflow. (PDF 531 kb)
Additional file 2: Appendix 1. Postextubation dysphagia screening form. (PDF 583 kb)
Additional file 3: Table S1. Questions and answers for the written test. (PDF 458 kb)
Additional file 4: Appendix 2. Nursing competency assessment form. (PDF 500 kb)
Additional file 5: Figure S2. Postextubation oral feeding and pneumonia outcomes. (PDF 445 kb)
Additional file 6: Table S2. Correlation between swallowing screen and oral feeding upon ICU discharge. (PDF 492 kb)

## Abbreviations

APACHE: Acute Physiology and Chronic Health Evaluation
ICU: Intensive care unit
LOS: Length of stay
NPS: Nurse-performed screening
SLP: Speech-language pathologist
CHF: Chronic heart failure
CKD: Chronic kidney disease
COPD: Chronic obstructive pulmonary disease
CLD: Chronic liver disease
IHD: Ischemic heart disease
IQR: Interquartile range
OSA: Obstructive sleep apnea
