# Supplementary material for: Nurse-performed screening for postextubation dysphagia: a retrospective cohort study in critically ill medical patients
Source: Crit Care. 2016 Oct 12;20:326. doi: 10.1186/s13054-016-1507-y (PMC5062851; doi:10.1186/s13054-016-1507-y)
Supplement: Additional file 2: Appendix 1. — Postextubation dysphagia screening form. (PDF 583 kb) [file 13054_2016_1507_MOESM2_ESM.pdf]

Supplemental Material

Nurse-Performed Screening for Post-Extubation Dysphagia: A Before-And-After Study in Critically-Ill Medical Patients

See KC[1,2], Peng SY[2], Phua J[1,2], Sum CL[3], Concepcion J[4]
[1]Division of Respiratory & Critical Care Medicine, University Medicine Cluster, National University Health System, Singapore
[2]Yong Loo Lin School of Medicine, National University of Singapore, Singapore
[3]Department of Nursing, National University Hospital, Singapore
[4]Department of Rehabilitation, National University Hospital, Singapore

Patient details
Please paste sticky label here

Post-Extubation Dysphagia Screening Form

National University Hospital

Day	1	2	3
Passed/ Failed			

Ward/ Bed No.: ______________________________

Date/Time: ______________________________

Qualified RN's Name & Signature: ______________________________

Patient successfully extubated/ off ventilator and planned for oral feeding (by Registrar & above)

→ NO →
- **DO NOT** proceed with screening
- Keep patient on non-oral feeding

↓ YES

Patient with tracheostomy

→ YES → (DO NOT proceed with screening; Keep patient on non-oral feeding)

↓ NO

Y	N	
☐	☐	Alert
☐	☐	Able to follow instructions
☐	☐	Able to be propped up in bed for feeding
☐	☐	Able to maintain SpO2 >90% without NIV

→ **No** on ANY item →
- Discontinue screening
- Keep patient on non-oral feeding
- Inform doctor
- Repeat screening the following morning before the doctor's rounds

↓ **Yes** on ALL items

Give patient 5 mls of water by teaspoon

Y	N	
☐	☐	Drooling
☐	☐	Choking
☐	☐	Coughing
☐	☐	Change in voice (gurgly, wet, more hoarse)

→ **Yes** on ANY items → (Discontinue screening; Keep patient on non-oral feeding; Inform doctor; Repeat screening the following morning before the doctor's rounds)

↓ **No** on ALL items

Give patient 60 mls of water by cup

Y	N	
☐	☐	Drooling
☐	☐	Choking
☐	☐	Coughing
☐	☐	Change in voice (gurgly, wet, more hoarse)

→ **Yes** on ANY items → (Discontinue screening; Keep patient on non-oral feeding; Inform doctor; Repeat screening the following morning before the doctor's rounds)

↓ **No** on ALL items

Inform Registrar & above.
If allowed to start oral feeding: FM diet for patients without dentition;
Chopped**,** Soft Diet or DOC for patients with adequate dentition)

→ RN to start oral feeding for patient and to supervise the first feed
