# Supplementary material for: Nurse-performed screening for postextubation dysphagia: a retrospective cohort study in critically ill medical patients
Source: Crit Care. 2016 Oct 12;20:326. doi: 10.1186/s13054-016-1507-y (PMC5062851; doi:10.1186/s13054-016-1507-y)
Supplement: Additional file 3: Table S1. — Questions and answers for the written test. (PDF 458 kb) [file 13054_2016_1507_MOESM3_ESM.pdf]

Supplemental Material

Nurse-Performed Screening for Post-Extubation Dysphagia: A Before-And-After Study in Critically-Ill Medical Patients

See KC[1,2], Peng SY[2], Phua J[1,2], Sum CL[3], Concepcion J[4]

[1]Division of Respiratory & Critical Care Medicine, University Medicine Cluster, National University Health System, Singapore
[2]Yong Loo Lin School of Medicine, National University of Singapore, Singapore
[3]Department of Nursing, National University Hospital, Singapore
[4]Department of Rehabilitation, National University Hospital, Singapore

e-TABLE 1. Questions and answers for the written test

No.	Question	Answer
1	This refers to difficulties in moving the food from the mouth down to the stomach through the esophagus	Dysphagia
2	Give two signs of aspiration	Coughing<br>Throat clearing<br>Choking<br>Desaturation
3	Give two complications of aspiration	Pneumonia<br>Dehydration<br>Malnutrition
4	Using the Massey Protocol, how many ml of water is given to the patient when no signs of aspiration were observed after drinking 5 ml of water?	60 ml
5	Give one exclusion criteria for the water swallow test	Patients on tracheostomy tube
6	Give one inclusion criteria for the water swallow test	Successfully Extubated<br>Planned for oral feeding (as advised by at least a Registrar)
7	Give one readiness criteria for the water swallow test	Alert<br>Cooperative<br>Able to sit up in bed<br>Maintain SpO2 90% and above without NIV
8	The patient passed the water swallow test. He has no teeth. What diet texture will you recommend this patient to start with?	Finely minced diet or blended diet
