# Supplementary material for: Nurse-performed screening for postextubation dysphagia: a retrospective cohort study in critically ill medical patients
Source: Crit Care. 2016 Oct 12;20:326. doi: 10.1186/s13054-016-1507-y (PMC5062851; doi:10.1186/s13054-016-1507-y)
Supplement: Additional file 4: Appendix 2. — Nursing competency assessment form. (PDF 500 kb) [file 13054_2016_1507_MOESM4_ESM.pdf]

Supplemental Material

Nurse-Performed Screening for Post-Extubation Dysphagia: A Before-And-After Study in Critically-Ill Medical Patients

See KC[1,2], Peng SY[2], Phua J[1,2], Sum CL[3], Concepcion J[4]

[1]Division of Respiratory & Critical Care Medicine, University Medicine Cluster, National University Health System, Singapore
[2]Yong Loo Lin School of Medicine, National University of Singapore, Singapore
[3]Department of Nursing, National University Hospital, Singapore
[4]Department of Rehabilitation, National University Hospital, Singapore

Nurse-Performed Screening for Post-Extubation Dysphagia

Nursing Competency Assessment Form

Name: ____ Designation: ____

Employee No.: ____________________________________ Ward No.: ____

S/N	Criteria	Test 1	Test 2	Test 3	Test 4	Test 5	Comments
**PREPARATION**							
1	Determines patient's identity (name and identity number)						
2	Determines from the team doctor (Registrar & above) that the patient is planned for oral feeding after extubation						
3	Checks if patient has tracheostomy tube						
4	Determines patient's ability to:						
	a. to remain alert for the screening						
	b. to cooperate with the screening						
	c. to be propped up in bed for the screening						
	d. maintain SPO2 >90& without non-invasive ventlation						
5	Prepares materials						
	a. teaspoon						
	b. water						
	c. measuring/ medicine cup						
	d. Dysphagia Screening Form						
	e. Pen						
6	Clearly explains to the patient:						
	a. the purpose of the screening						
	b. the test procedures						
**PERFORMANCE**							
7	Positions patient appropriately						
8	Commences dysphagia screening by giving patient 5 ml water by spoon						
9	Identifies signs of aspiration observed						
	a. drooling						
	b. choking						
	c. gurgling sounds						
	d. coughing						
10	If signs of aspiration present:						
	a. discontinues the screening immediately						
	b. Aware of next steps (keeps the patient NBM, informs the doctor, await further advise from doctors)						

11	If no signs of aspiration present, proceeds with the dysphagia screening by giving patient 60 ml water by cup.						
12	Identifies signs of aspiration observed						
	a. Drooling						
	b. choking						
	c. gurgling sounds						
	d. coughing						
13	If signs of aspiration present:						
	a. discontinues the screening immediately						
	b. aware of next steps (keeps the patient NBM, informs the doctor, await further advice from doctors)						
14	If no signs of aspiration present:						
15	a. informs doctors of screening results and await further advice.						
	b. appropriate diet recommendations given for patients who are appropriate to commence oral feeding (dentate: chopped diet; edentate: finely minced diet)						
**FOLLOW UP**							
16	For patients who are to commence oral feeding:						
	a. supervises first meal to observe for signs of aspiration						
	b. documents supervision of the first meal						
17	For patient who fail the dysphagia screening test and requires referral to speech therapist: informs doctor to order referral						
18	Files Dysphagia Screening Form in the appropriate section of the case notes						
19	Documents findings in the case notes						

Overall result	**Competency ACHIEVED / NOT ACHIEVED**
Assessor’s Name/ Designation	
Signature	
Date	
