# Supplementary material for: Nurse-performed screening for postextubation dysphagia: a retrospective cohort study in critically ill medical patients
Source: Crit Care. 2016 Oct 12;20:326. doi: 10.1186/s13054-016-1507-y (PMC5062851; doi:10.1186/s13054-016-1507-y)

Supplemental Material

Nurse-Performed Screening for Post-Extubation Dysphagia: A Before-And-After Study in Critically-Ill Medical Patients

See KC[1,2], Peng SY[2], Phua J[1,2], Sum CL[3], Concepcion J[4]

[1]Division of Respiratory & Critical Care Medicine, University Medicine Cluster, National University Health System, Singapore
[2]Yong Loo Lin School of Medicine, National University of Singapore, Singapore
[3]Department of Nursing, National University Hospital, Singapore
[4]Department of Rehabilitation, National University Hospital, Singapore

e-FIGURE 2. Post-extubation oral feeding and pneumonia outcomes

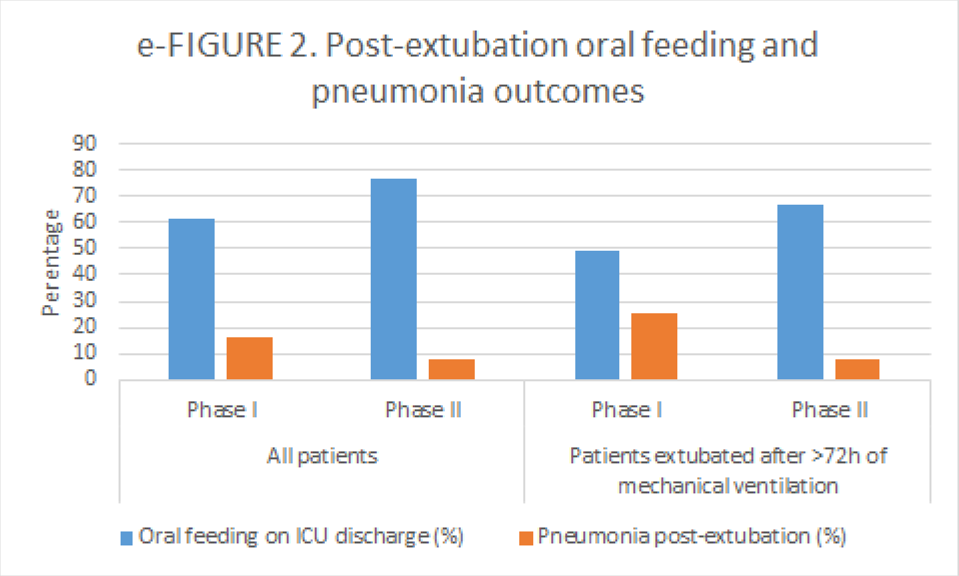

Supplement: Additional file 5: Figure S2. — Postextubation oral feeding and pneumonia outcomes. (PDF 445 kb) [file 13054_2016_1507_MOESM5_ESM.pdf]
