# Supplementary material for: Nurse-performed screening for postextubation dysphagia: a retrospective cohort study in critically ill medical patients
Source: Crit Care. 2016 Oct 12;20:326. doi: 10.1186/s13054-016-1507-y (PMC5062851; doi:10.1186/s13054-016-1507-y)
Supplement: Additional file 6: Table S2. — Correlation between swallowing screen and oral feeding upon ICU discharge. (PDF 492 kb) [file 13054_2016_1507_MOESM6_ESM.pdf]

Supplemental Material

Nurse-Performed Screening for Post-Extubation Dysphagia: A Before-And-After Study in Critically-Ill Medical Patients

See KC[1,2], Peng SY[2], Phua J[1,2], Sum CL[3], Concepcion J[4]

[1]Division of Respiratory & Critical Care Medicine, University Medicine Cluster, National University Health System, Singapore
[2]Yong Loo Lin School of Medicine, National University of Singapore, Singapore
[3]Department of Nursing, National University Hospital, Singapore
[4]Department of Rehabilitation, National University Hospital, Singapore

e-TABLE 2. Correlation between swallowing screen and oral feeding on ICU discharge

	Passed swallowing screen	Did not pass swallowing screen[a]	Total
**On oral feeding on ICU discharge**	136	8	144
**Not on oral feeding on ICU discharge**	7	36	43
**Total**	143	44	187

Overall correlation = 172/187 x 100% = 92.0%

[a] Includes patients who failed the swallowing screen overall and patients who missed all three days of screening
